# Optimization of an Image-Guided Laser-Induced Choroidal Neovascularization Model in Mice

**DOI:** 10.1371/journal.pone.0132643

**Published:** 2015-07-10

**Authors:** Yan Gong, Jie Li, Ye Sun, Zhongjie Fu, Chi-Hsiu Liu, Lucy Evans, Katherine Tian, Nicholas Saba, Thomas Fredrick, Peyton Morss, Jing Chen, Lois E. H. Smith

**Affiliations:** 1 Department of Ophthalmology, Boston Children’s Hospital, Harvard Medical School, Boston, Massachusetts, United States of America; 2 Department of Ophthalmology, Sichuan Provincial Hospital and Sichuan Academy of Medical Science, Chengdu, Sichuan, People’s Republic of China; Indiana University College of Medicine, UNITED STATES

## Abstract

The mouse model of laser-induced choroidal neovascularization (CNV) has been used in studies of the exudative form of age-related macular degeneration using both the conventional slit lamp and a new image-guided laser system. A standardized protocol is needed for consistent results using this model, which has been lacking. We optimized details of laser-induced CNV using the image-guided laser photocoagulation system. Four lesions with similar size were consistently applied per eye at approximately double the disc diameter away from the optic nerve, using different laser power levels, and mice of various ages and genders. After 7 days, the mice were sacrificed and retinal pigment epithelium/choroid/sclera was flat-mounted, stained with Isolectin B4, and imaged. Quantification of the area of the laser-induced lesions was performed using an established and constant threshold. Exclusion criteria are described that were necessary for reliable data analysis of the laser-induced CNV lesions. The CNV lesion area was proportional to the laser power levels. Mice at 12-16 weeks of age developed more severe CNV than those at 6-8 weeks of age, and the gender difference was only significant in mice at 12-16 weeks of age, but not in those at 6-8 weeks of age. Dietary intake of omega-3 long-chain polyunsaturated fatty acid reduced laser-induced CNV in mice. Taken together, laser-induced CNV lesions can be easily and consistently applied using the image-guided laser platform. Mice at 6-8 weeks of age are ideal for the laser-induced CNV model.

## Introduction

Age-related macular degeneration (AMD) is a major cause of blindness and vision impairment in the elderly [1,2]. Neovascular AMD is characterized by choroidal neovascularization (CNV), with blood vessels from the choriocapillaris penetrating through Bruch's membrane into the normally avascular subretinal space [3,4]. Although only ~10% of AMD patients develop neovascular AMD, it accounts for ~90% of AMD-associated vision loss with deterioration of central vision that impacts the daily activities of affected patients [1,5]. Developing a reproducible model that mimics neovascular AMD is needed to study this disease.

*In vitro* endothelial cell culture models of CNV lack complex *in vivo* cellular interactions with photoreceptors, retinal pigment epithelium, pericytes, inflammatory cells and glial cells [6]. A laser-induced *in vivo* model of CNV, first described in 1979 [7], uses photocoagulation to disrupt Bruch’s membrane, inducing the growth of new choroidal vessels into the subretinal area. This model is similar to neovascular AMD in that vessels arise from the choroid. However it differs from AMD as it is a wounding model unlike neovascular AMD that is initiated with aging changes. The laser-induced CNV model has been successful in predicting the clinical efficacy of anti-vascular endothelial growth factor (VEGF) therapy for neovascular AMD [8]. Although it is frequently used to study CNV and evaluation of anti-angiogenic drugs *in vivo*, it has been limited in predicting efficacy of drugs other than those involving the VEGF pathway [9]. While also available in rats and monkeys [7,8], this model in mice can be used in transgenic animals to explore the molecular mechanisms of CNV formation [10]. Optimizing the parameters of the CNV model will make it more reproducible and extend its use.

A slit lamp is often used to administer laser photocoagulation [2,9,11]. This system has some limitations including difficulty in administering consistent laser burns. There is an alternative laser system available, the Micron IV platform guided by real-time fundus imaging. We optimized laser power, the age and sex of mice, and lesion analysis methods to create reproducible CNV lesions using this real-time fundus image-guided laser system. We then assessed the effect of dietary intervention with omega-3 unsaturated fatty acid on CNV using our optimized parameters. We proposed a set of guidelines to help produce consistent CNV lesions and minimize the number of lesions with bleeding which will add to the reproducibility and reliability of the laser-induced CNV model commonly used for neovascular AMD research.

## Materials and Methods

### Mice

C57BL/6J mice (Jackson Laboratory, Bar Harbor, ME) were treated in accordance with the Association for Research in Vision and Ophthalmology Statement for the Use of Animals in Ophthalmic and Vision Research. All animal studies were performed according to the protocols reviewed and approved by the Institutional Animal Care and Use Committee at Boston Children’s Hospital.

### Laser Photocoagulation

Mice were anesthetized with a mixture of xylazine (6 mg/kg) and ketamine (100 mg/kg), and pupils were dilated with topical drops of Cyclomydril (Alcon Laboratories, Fort Worth, TX). Two minutes after pupil dilation, lubricating eye drops (Alcon Laboratories) were applied to the cornea. The fundus was viewed with an imaging camera, and laser photocoagulation was induced using the image-guided laser system (Micron IV, Phoenix Research Laboratories, Pleasanton, CA). The fundus image as well as the aiming beam can be observed on the monitor screen. Four laser burns at equal distance from the optic nerve were induced one by one in each eye by a green Argon laser pulse with a wavelength of 532 nm, a fixed diameter of 50 μm, duration of 70 ms, and varying power levels from 180 mW to 360 mW. If necessary, an orienting laser shot can also be generated approximately three times of the diameter of the optic nerve to help determine the relative positions of the lesions in an eye. After laser photocoagulation, the eyes were gently rinsed with sterile saline to remove the lubricating eye drops and treated with an antibiotic ointment, erythromycin (Fougera, Melville, NY). Mice were then placed on a pre-warmed warming plate at 35°C after the laser treatment until they awakened.

### Optical Coherence Tomography (OCT)

Mouse pupils were dilated with Cyclomydril drops after the mice were anesthetized by the xylazine-ketamine mixture described above. Spectral domain optical coherence tomography (SD-OCT) with guidance of bright-field live fundus image was performed using the image-guided OCT system (Micron IV, Phoenix Research Laboratories) according to the manufacturer’s instruction and using the vendor’s image acquisition software to generate bright field images, angiograms, and OCT scans.

### Fundus Fluorescein Angiography (FFA)

FFA to determine leakage (not to determine lesion size) was performed with the retinal imaging microscope (Micron IV, Phoenix Research Laboratories) 6 days after laser photocoagulation. Mice were anesthetized, pupils dilated, and intraperitoneally injected with fluorescein AK-FLUOR (Akorn, Lake Forest, IL) at 5 μg/g body weight. Fluorescent fundus images were taken with the retinal imaging microscope at 5 and 10 minutes after fluorescein injection. The fluorescent intensity of CNV lesions was graded using ImageJ (National Institutes of Health, Bethesda, MD) by masked researchers [12], and the difference of fluorescent intensity between 5 and 10 minute images were recorded as an indicator of CNV vascular leakage.

### Retinal pigment epithelium/Choroid/Sclera Flat-mount, Imaging and Quantification

Mice were euthanatized 7 days after laser photocoagulation. Eyes were immediately enucleated and fixed with 4% paraformaldehyde (Sigma-Aldrich, St. Louis, MO) in PBS for 1 hour at room temperature. For histology study, eyes were embedded in Tissue-Tek O.C.T. Compound (Sakura, Torrance, CA), sectioned, and stained with hematoxylin and eosin [13]. For flat-mounts, the posterior eye cups consisting of the retinal pigment epithelium/choroid/sclera were dissected and permeabilized with Triton X-100 (0.1%, Thermo Fisher Scientific, Tewksbury, MA) in phosphate buffered saline (PBS, Life Technologies, Grand Island, NY) for 1 hour at room temperature. The CNV lesions were stained with Isolectin B4 (IB4, 10 μg/ml, Life Technologies) at room temperature overnight. After washing with PBS three times, 15 min each, the posterior eye cups were flat-mounted onto slides (Thermo Fisher Scientific) with the scleral side down in SlowFade anti-fade mounting medium (Life Technologies). Both the hematoxylin and eosin, and fluorescent images were taken with the AxioCam MRm and AxioObserver.Z1 microscope (Zeiss, Peabody, MA) and the areas of CNV lesions were quantified in masked fashion [9].

### Statistics

Data are presented as mean ± SEM. Student’s t test was used to compare 2 groups of samples. For more than 2 groups of samples, one-way ANOVA was performed using Prism 6 (GraphPad, San Diego, CA). p ≤ 0.05 was considered as statistically significant.

## Results

### The image-guided laser photocoagulation system produced consistent leaky CNV lesions

**Fig 1:**
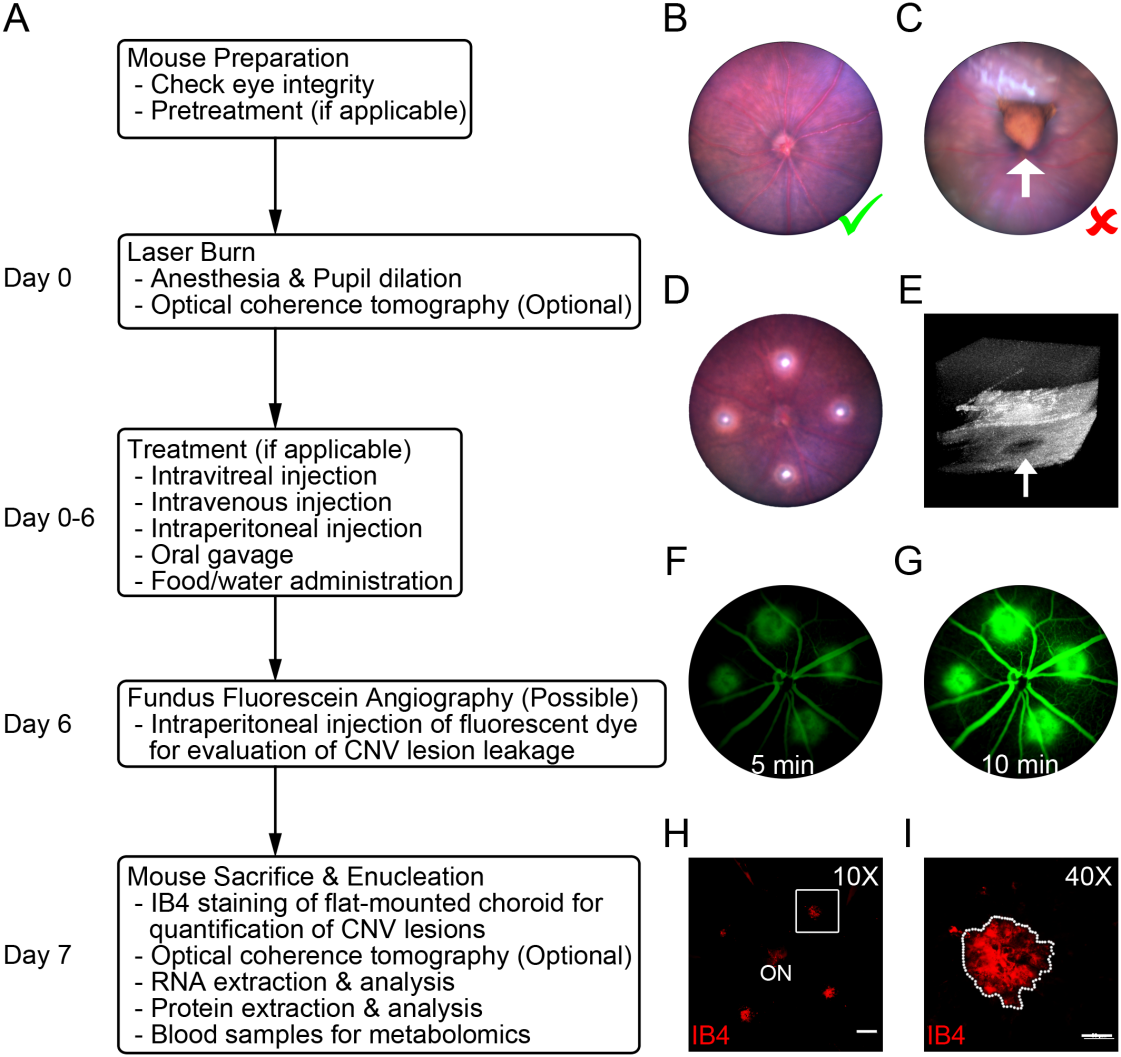
Experimental Flow Chart of the Image-Guided Laser-Induced CNV Model and Data Collection. (A) Overview of the procedure for CNV induction involving mouse preparation and followed by experimental treatment, sample preparation and analysis. (B) Representative image of normal fundus (Green check mark). (C) Representative image of anomalous structure (white arrow) in the eye, which is not suitable for laser photocoagulation (Red X). (D) Representative image of normal fundus with 4 laser burns shown as bright white spots. (E) Representative image of a successful laser burn (white arrow) with 3D OCT. (F&G) Representative ocular FFA images at 5 and 10 minutes after the injection of fluorescent dye at day 6 after laser burn. (H) Representative images of flat-mounted choroid with IB4 staining at day 7 after laser photocoagulation. Scale bar: 200 μm. ON, optic nerve. (I) Higher magnification of the laser-induced CNV lesion highlighted in panel H. Scale bar: 50 μm.

C57BL/6J mice were used for all experiments, because only pigmented mice absorb laser energy well and respond reliably to laser burns in the eye. The general procedure of laser-induced CNV induction involves mouse anesthesia, mouse positioning, laser burn, (optional OCT and FFA), eye dissection, choroid staining and imaging, and CNV lesion quantification (Fig 1A). Most operations, from eye integrity check and laser photocoagulation to OCT and FFA, were performed using the integrated platform (Micron IV). Only intact eyes (Fig 1B) without observable structural or morphological abnormalities were used for the laser-induced CNV model. Eyes with anomalous structures (Fig 1C), cataract or visible defects of the cornea or fundus were excluded. After anesthesia and pupil dilation, 4 laser burns per eye were induced using a green Argon laser focusing on the fundus (Fig 1D). Optional OCT immediately after laser photocoagulation may be used to confirm the success of the laser burn with visible rupture of Bruch’s membrane (Fig 1E). Mice with or without treatment can be subjected to FFA to evaluate the levels of vascular leakage from CNV lesions 6 days after laser burn (Fig 1F & 1G). The *in vivo* retinal structure may also be examined by OCT, if applicable, to determine the cross-sectional area of CNV lesions 7 days after laser burns. To measure the surface area of CNV lesions, the fluorescence-stained retinal pigment epithelium/choroid/sclera flat-mounts were imaged (Fig 1H & 1I) and quantified by researchers masked to treatment. The choroidal CNV samples may also be analyzed for RNA or protein. We found that image-guided laser photocoagulation is capable of producing consistent CNV lesions that can be used to evaluate the effects of interventions on size and permeability.

### Even focus was essential for producing consistent laser photocoagulation and CNV lesions

**Fig 2:**
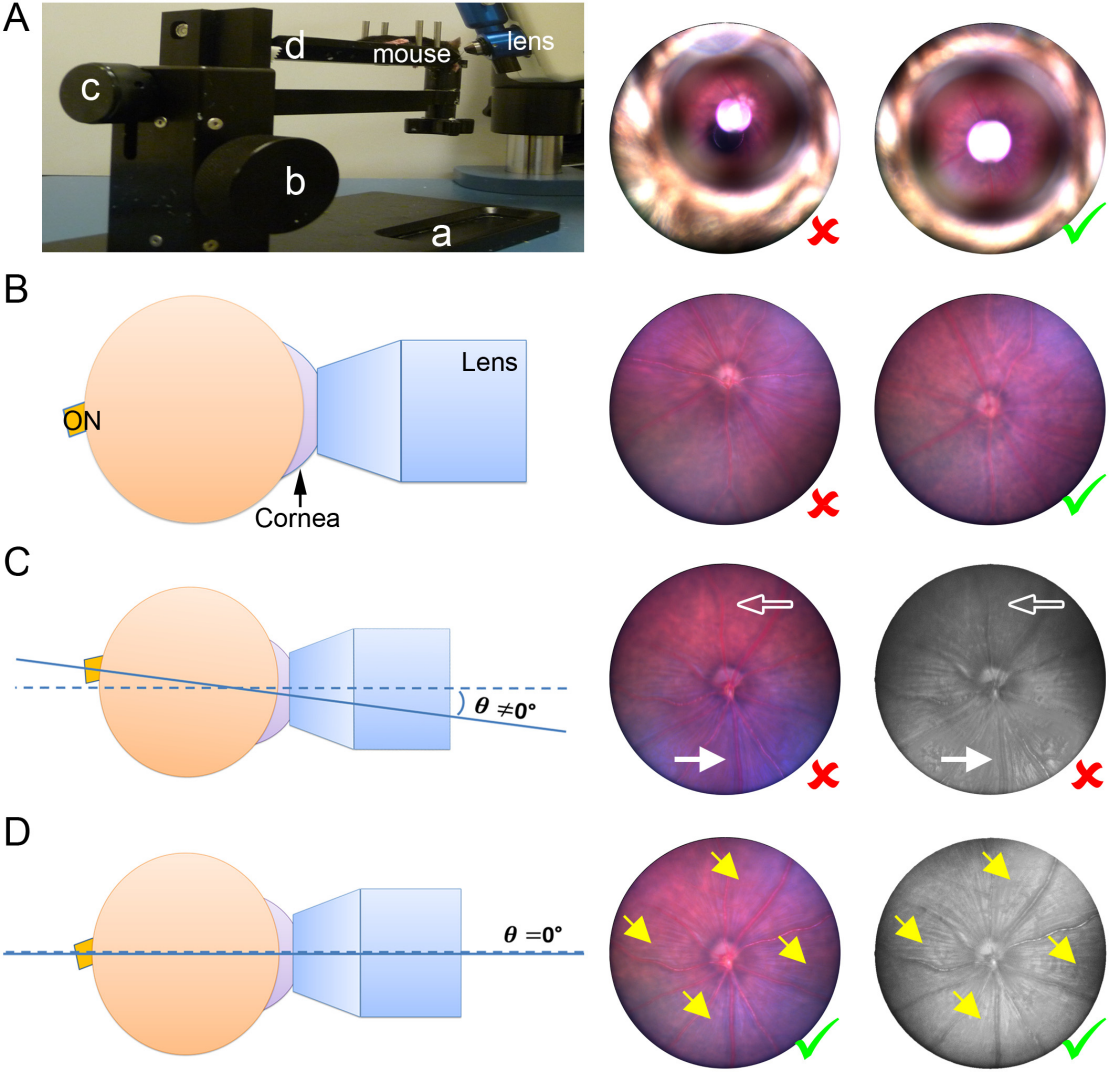
Focus Adjustment Using Micron IV. (A) Prior to lens contact with the eye, the ON was positioned in the center of the vision by moving the mouse support (a) and adjusting the height through knob b. (B) After lens contact with cornea, the ON was re-positioned in the center of the vision by fine adjustment through knob c and mouse platform (d). (C) Demonstration of representative incorrect alignment showed retinal ON fibers unevenly in the vision with the bottom half (solid arrow) much clearer than the upper half (hollow arrow), indicating the camera axis was not aligned with the eye axis. *θ*, the intersection angle between the eye and camera axises. (D) Even radial reflection of retinal ON fibers (yellow arrows) in all of the 4 quadrants indicated an ideal alignment (*θ* = 0) of the eye axis with the camera axis, which is critical to induce consistent and reliable laser photocoagulation.

We found that one key aspect of generating reliable and consistent CNV lesions with the image-guided laser system is the initial adjustment of focus. First, the lens should be positioned approximately 5 mm away from the cornea of mouse eyes where the major retinal vessels can be clearly observed by adjusting the lens focus. The optic nerve should be positioned in the center of the visual field by adjusting the position and height of the mouse holder (Fig 2A). Next, the lens should be slowly advanced until it gently contacts the cornea and the optic nerve should be re-positioned in the center of the view field by fine positional adjustment (Fig 2B). One crucial adjustment is to align the axis of both the mouse eye and the lens, for the subsequent best laser alignment. The eye axis and the lens axis are aligned when the reflection of the retinal nerve fibers is evenly bright and clear in all directions (Fig 2C & 2D). The precise alignment together with accurate focus is the essential prerequisite for consistent laser photocoagulation in the eye.

### Formation of a vaporization bubble indicates successful laser photocoagulation

**Fig 3:**
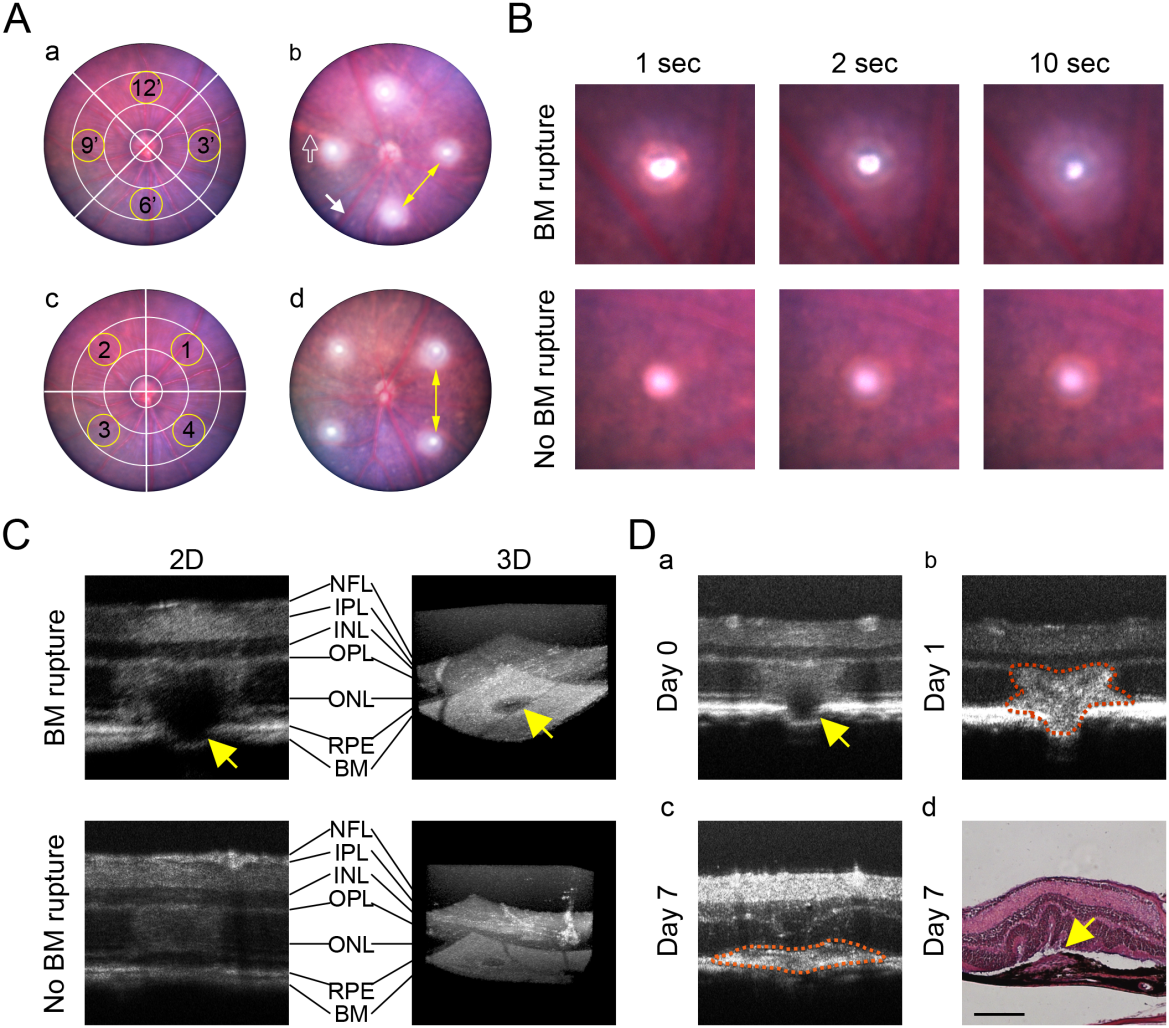
Suggested Retinal Positions of Laser Photocoagulation and Indicator of Successful Rupture of Bruch’s Membrane. (A) Four laser burns per eye were applied at 3, 6, 9 and 12 o’clock (a) or in 4 individual quadrants (c) approximately double the disc diameter of the optic nerve away from it, which was between the 2 circles. Main retinal vessels (solid arrow in b) and choroid vessels (empty arrow in b) should be avoided to prevent severe breeding. The distance between laser burns (yellow arrows in b&d) should be at least double the optic nerve diameter. (B) A successful laser-induced rupture of Bruch’s membrane (BM) was identified by the appearance of a vaporization bubble and haze area around the lesion right after laser photocoagulation (upper panels). If the Bruch’s membrane was not ruptured, vaporization bubble or haze area would not occur (lower panels). (C) The rupture of BM (yellow arrows) induced by laser burn was confirmed by both 2D cross-sectional OCT scan and 3D reconstructed OCT image. NFL: nerve fiber layer; IPL: inner plexiform layer; INL: inner nuclear layer; OPL: outer plexiform layer; ONL: outer nuclear layer; RPE: retinal pigment epithelium. (D) Cross-sectional OCT scans of the lesion showing the rupture of BM at day 0 (yellow arrow in a), a typical butterfly-like shape of retinal hyper-reflectivity at day 1 (b), choroidal fibro-vascular tissue (marked by red dot line) formation at day 7 (c) and a typical section of laser-induced CNV lesion (yellow arrow in d) stained with hematoxylin and eosin at day 7 after laser photocoagulation. Scale bar: 200 μm.

Once the fundus is in focus, both the retinal major vessels (bright red in the visual field) and the large-size choroidal vessels (pink) can be observed clearly. Four laser burns per eye should be generated at equal distance from the optic nerve (which optimally is approximately twice of the diameter of the optic nerve) at the 3, 6, 9 and 12 o’clock positions or in the center of 4 individual retinal quadrants (Fig 3A). The distance between laser burns must be at least double the diameter of the optic nerve to avoid fusion of lesions. Major retinal and choroidal vessels should be avoided to prevent potential bleeding (Fig 3A). The formation of a vaporization bubble immediately after laser photocoagulation indicates the success of a laser burn, which correlates with a rupture of Bruch’s membrane (Fig 3B). Both 2 dimensional (2D) and 3D OCT images may be used to confirm the rupture of Bruch’s membrane (Fig 3C). If OCT is used, Bruch’s membrane rupture can be observed in the images showing the typical butterfly-like structure at day 1 and newly formed subretinal CNV at day 7 (Fig 3D). These results suggest that the laser photocoagulation delivered by the image guided laser system is capable of generating CNV lesions with comparable morphological features (vaporization bubble and butterfly-like structure) as the conventional slit lamp system [2,14].

### Exclusion criteria are necessary for evaluation of laser-induced CNV lesions

The laser-induced CNV model in mice has been often characterized as variable and inconsistent [9]. Establishing a set of consistent exclusion criteria is necessary for ensuring reliable data analysis. In a typical study, 10 mice per group with 4 lesions per eye would optimally provide 80 data points for each experimental condition. To account for data or mouse loss, including (1) cataract and corneal epithelial edema before laser photocoagulation, (2) unsuccessful laser burn without Bruch’s membrane rupture (Fig 3B & 3C), (3) odd lesion shape due to mouse movements during laser induction, (4) death of mice post-laser treatment, or (5) damage of the CNV lesions during tissue dissection and processing, more mice may be needed and should be considered in a power analysis to account for an anticipated intervention effect [9].

**Fig 4:**
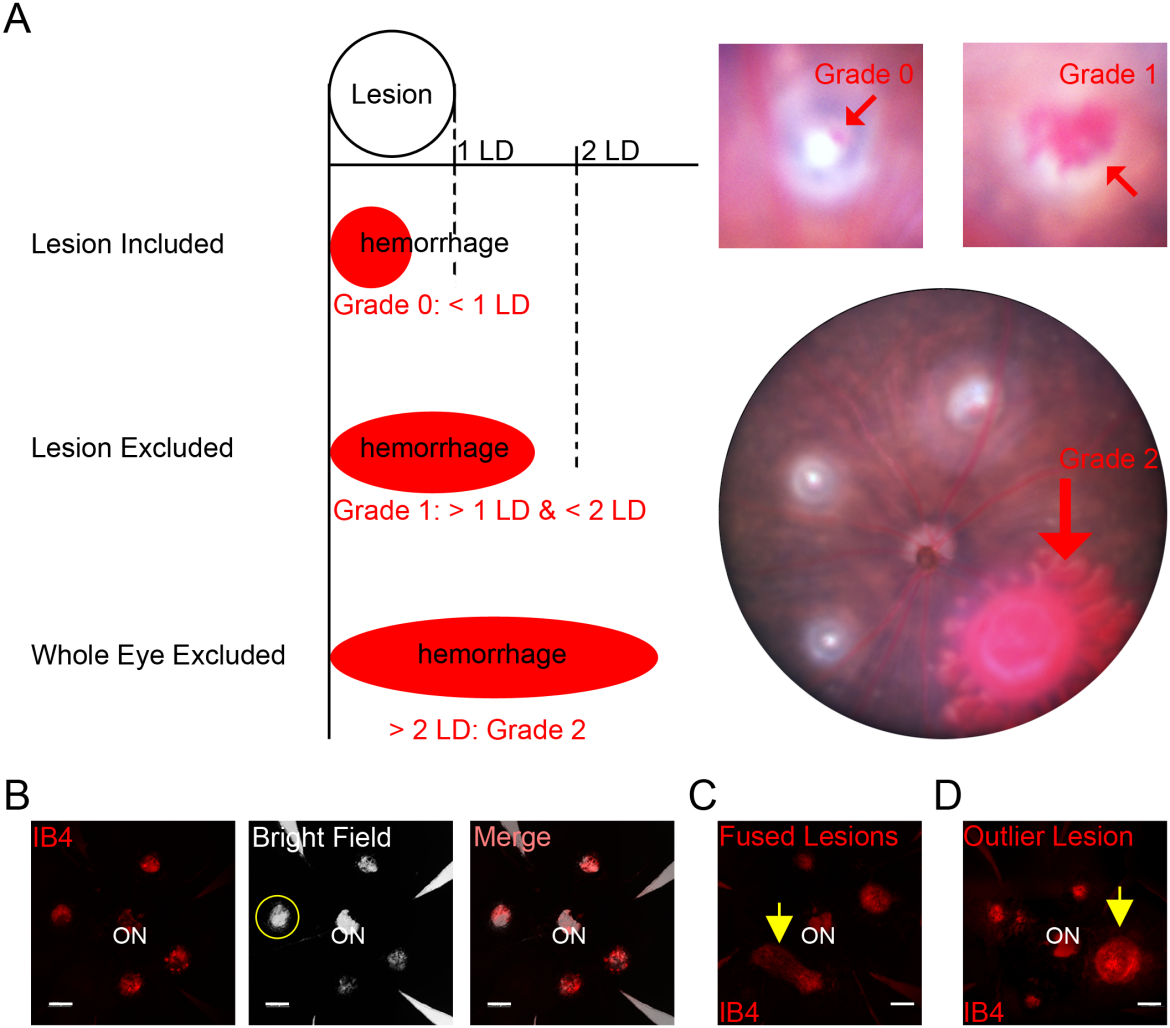
Exclusion Criteria for the Laser-Induced Lesions. (A) Laser-induced choroidal hemorrhages were graded as follows: Grade 0, the major axis of the bleeding area was smaller than the diameter of the laser-induced lesion; Grade 1, the major axis of the bleeding area was bigger than the diameter of the lesion but smaller than 2 times of the lesion diameter (LD); and Grade 2, the major axis of the bleeding area was bigger than 2 LD. Lesions with Grade 0 bleeding were included, lesions with Grade 1 bleeding were excluded and any eyes with Grade 2 bleeding were excluded. (B) Lesions with choroidal damage (yellow circle in bright field image) were excluded. Scale bar: 200 μm. (C) Fused lesions (yellow arrow) were excluded. Scale bar: 200 μm. (D) Outlier lesions (yellow arrow) with more than 5 times larger than the mean area of the lesions in the same eye were excluded. Scale bar: 200 μm.

To accurately evaluate the laser-induced CNV, some lesions should be excluded. Severe hemorrhages will cause much larger CNV lesions, whereas choroidal damage will yield a CNV lesion much smaller than the fellow CNV lesions in the same eye. First, choroidal hemorrhages encroaching on the lesion should be analyzed and classified carefully (Fig 4A): (1) if the diameter of bleeding area is less than that of the lesion, the lesion (Grade 0) will be eligible for inclusion of analysis (2) if the diameter of bleeding area is more than that of the lesion but less than 2 times of the lesion diameter, the lesion (Grade 1) should be excluded from quantification (3) if the diameter of bleeding area is more than 2 times the lesion diameter (Grade 2), all lesions in the same eye should be excluded from analysis. Second, excessive laser burns that damage not only Bruch’s membrane but also the choroid and retinal pigment epithelium should be excluded. These excessive burns can be seen clearly as a solid “hole” in the bright field of choroid imaging (Fig 4B). Lesions should also be excluded if (1) the lesion is fused with another lesion (Fig 4C), (2) the lesion is either more than 5 times larger than the mean of the lesions under the same experimental conditions (Fig 4D) [9], or (3) the lesion is the only one eligible for statistical analysis among all lesions in an eye.

### The CNV lesion area is proportional to the laser power levels

Previous studies indicate that the optimal time to measure the area of CNV lesion is at day 7 or day 14 after photocoagulation [2], and that there is no significant difference between lesion area at day 7 and 14. Therefore, to economize time and costs, we analyzed the lesion area at day 7 for all experiments.

**Fig 5:**
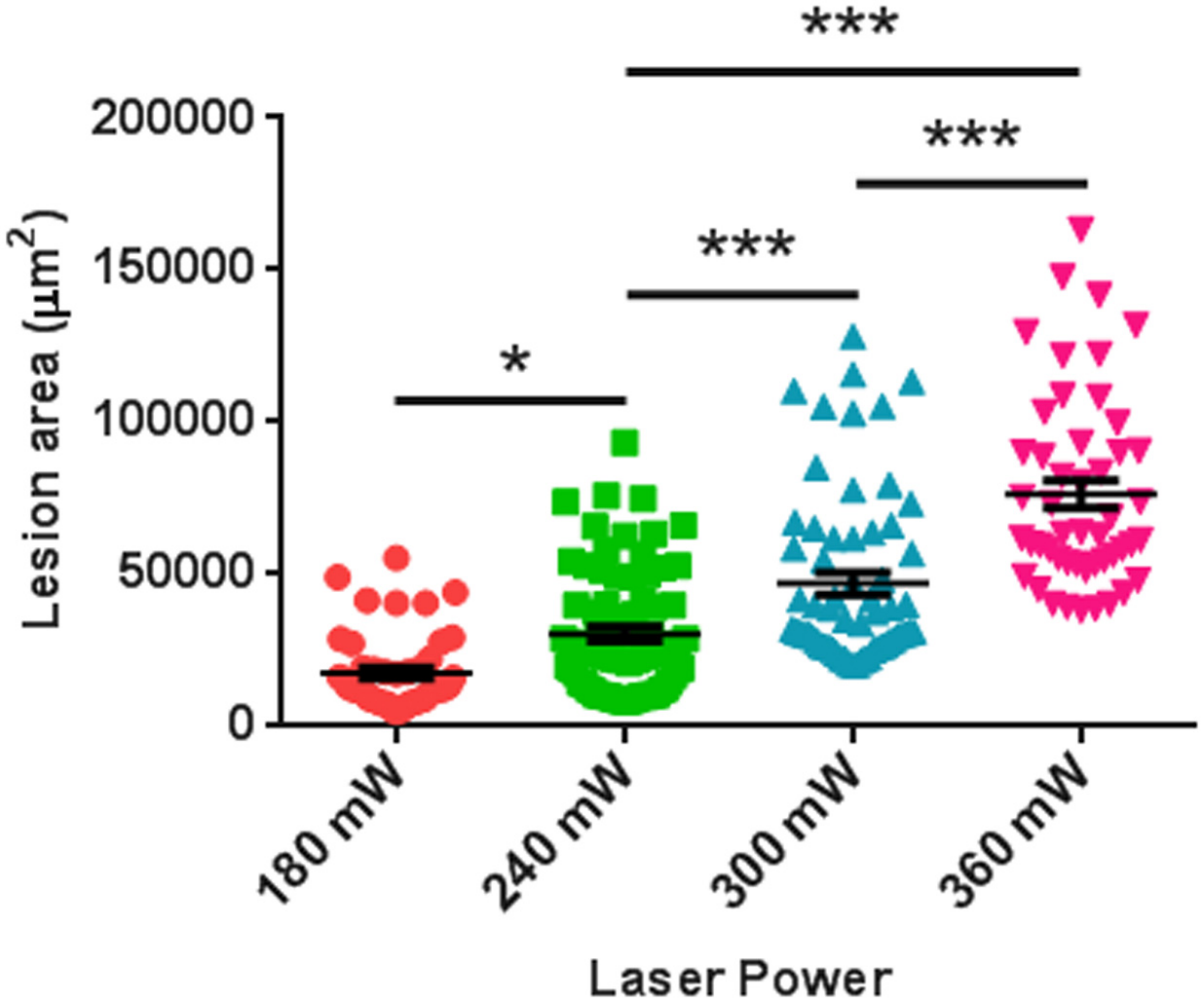
The Area of Lesions Was Positively Correlated to the Power Levels of Laser. Laser photocoagulation was induced with different levels of laser power in C57BL/6 mice using Micron IV. The area of lesions was quantified in flat-mounted choroids with IB4 staining 7 days after laser injection. n = 10 mice/group. * p < 0.05; *** p < 0.001.

Laser power from 180 mW to 360 mW with identical duration of 70 ms and wavelength of 532 nm was used for photocoagulation. The percentages of quantifiable lesions are shown in Table 1. The area of CNV lesions was positively correlated to the laser power level (Fig 5 and Table 2). We suggest that 240 mW is the optimal laser power level for laser photocoagulation in C57BL /6J mice using the Micron IV laser system. Lower laser power may lead to less successful Bruch’s membrane rupture, and higher laser power causes more bleeding, more choroidal damage, more fused lesions, and higher variation in lesion area.

**Table 1:** Percentages of Lesion Types with Different Laser Power Levels. BM, Bruch’s membrane.

Lesion Type (%)	180 mW	240 mW	300 mW	360 mW
**No BM Rupture**	20 (27.8%)	2 (2.5%)	1 (1.32%)	0 (0%)
**Lesion Included**	46 (63.9%)	75 (93.8%)	63 (82.9%)	49 (64.5%)
**Bleeding (G1&G2)**	0 (0%)	1 (1.25%)	5 (6.58%)	14 (18.4%)
**Choroidal Damage**	0 (0%)	0(0%)	2 (2.63%)	5 (6.58%)
**Fused Lesion**	0 (0%)	0 (0%)	2 (2.63%)	4 (5.26%)
**Outlier Lesion**	6 (8.33%)	2 (2.5%)	3 (3.95%)	4 (5.26%)
**Total Shots/Total Mice**	72/10	80/10	76/10	76/10

**Table 2:** Number of CNV Lesions, Mean Area CNV, SEM, SD and % Lesion Area Relative to Area at 240 mW Laser Power.

Laser Power	180 mW	240 mW	300 mW	360 mW
**Number of CNV Lesions**	46	75	63	49
**Mean Area CNV (μm^2^)**	17627.4	30433.0	47067.9	76326.2
**SEM**	12582.2	20368.9	28330.5	31594.9
**SD**	1855.14	2352.00	3569.30	4513.55
**% Lesion Area Relative to Area at 240 mW**	57.9	100	155	251

### Mice at 6–8 weeks of age are ideal for the laser-induced CNV model

**Fig 6:**
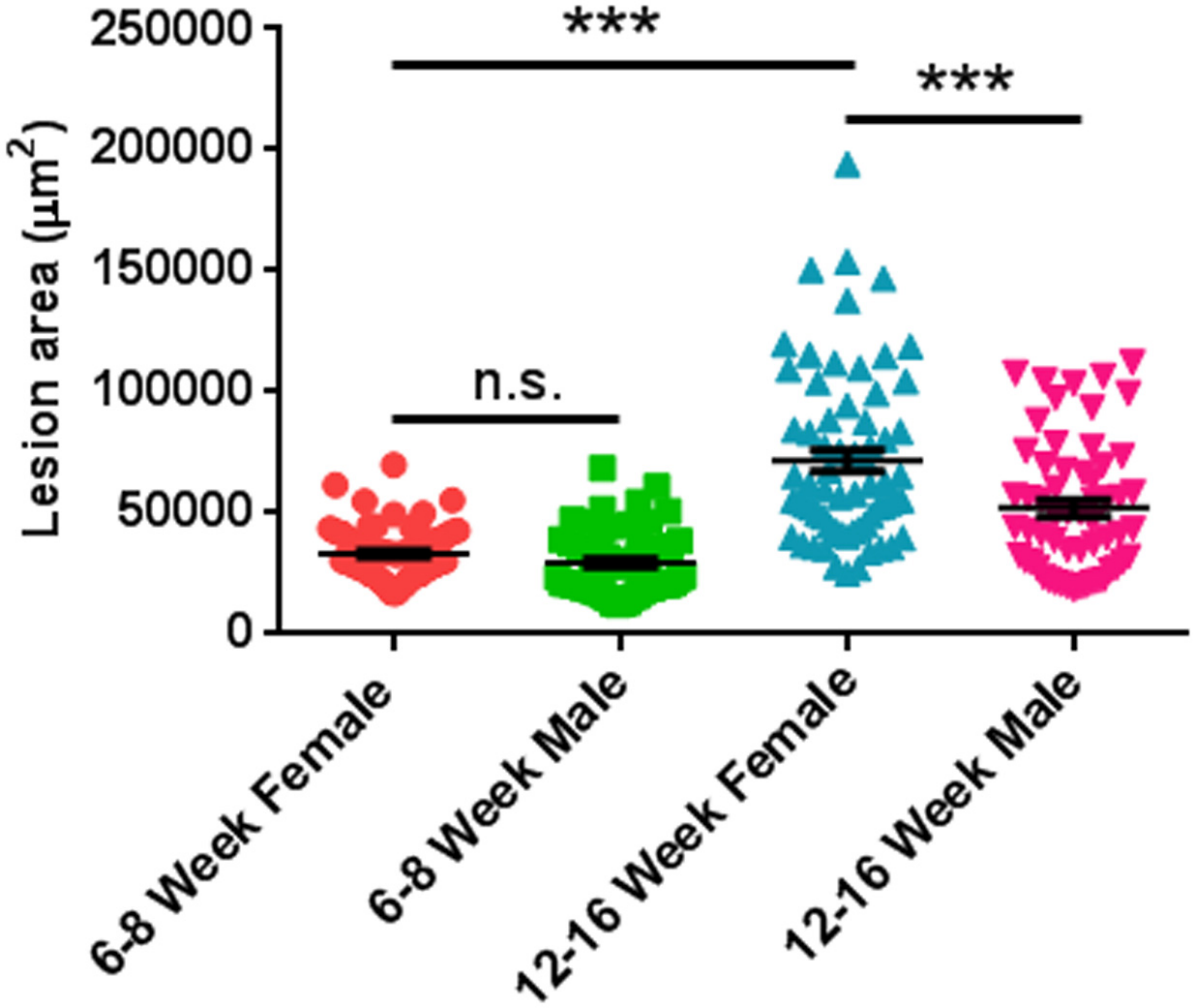
Gender Had Little Effect on CNV Lesion Area in Younger Mice. Laser photocoagulation was induced in C57BL/6 mice of both genders at 6–8 or 12–16 weeks age using Micron IV. The area of lesions was quantified in flat-mounted choroids with IB4 staining 7 days after laser injection. n = 10 mice/group. n.s. not significant; *** p < 0.001.

Previous studies suggest that both gender and age of animals influence the outcome of laser-induced CNV [11,15,16]. To clarify how these parameters affect the area of laser-induced CNV lesions, we assessed 4 different groups of mice with different combinations of age and gender: (1) female mice weighing 15–20 g at 6–8 weeks of age; (2) male mice weighing 18–23 g at 6–8 weeks of age; (3) female mice weighing 23–28 g at 12–16 weeks of age; and (4) male mice weighing 30–35 g at 12–16 weeks of age. We find that the older mice at 12–16 weeks of age develop more severe CNV than the younger mice at 6–8 weeks of age in both genders, and the gender difference was only significant in the older mice, but not in the younger mice. Especially noteworthy, the older female mice developed significantly larger CNV lesions than both older male and younger female mice (Fig 6 and Table 3). In addition, compared with the younger mice, the lesion area in the older mice had increased variation. These data suggest that mice at 6–8 weeks of age of both genders can be used most reproducibly for the laser-induced CNV model.

**Table 3:** Number of CNV Lesions, Mean Area CNV, SEM, SD and % Lesion Area Relative to Area of 6–8 Week Male Mice.

Mice	6–8 Week Female	6–8 Week Male	12–16 Week Female	12–16 Week Male
**Number of CNV Lesions**	67	58	66	60
**Mean Area CNV (μm^2^)**	33202.5	29445.4	71770.9	52104.9
**SEM**	10835.0	13059.7	35599.2	26895.8
**SD**	1323.70	1714.82	4381.96	3472.23
**% Lesion Area Relative to Area of 6–8 Week Male**	113	100	244	177

### Dietary intake of omega-3 long-chain polyunsaturated fatty acid reduced laser-induced CNV in mice

**Fig 7:**
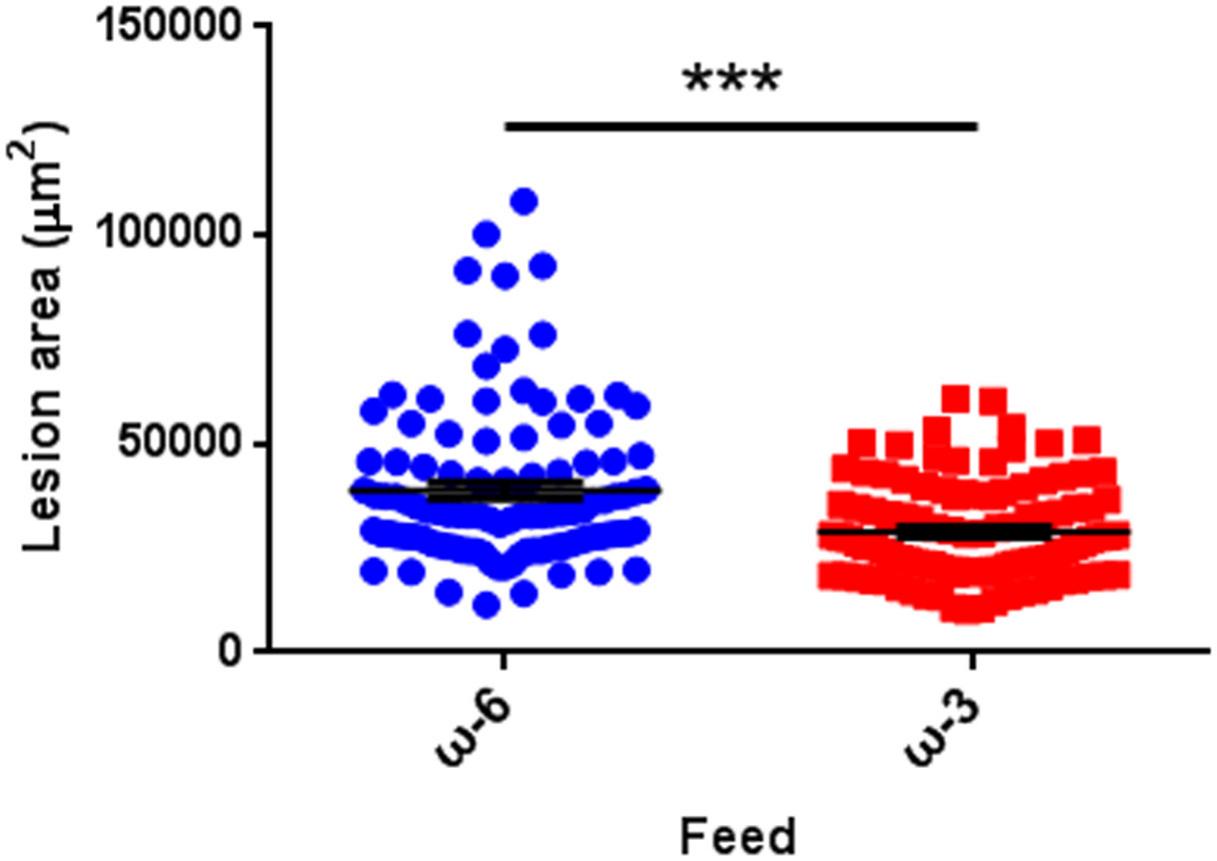
Dietary Intake of Omega-3 Polyunsaturated Fatty Acid Reduced CNV. C57BL/6 mice were fed with omega-6 (ω-6) or omega-3 (ω-3) polyunsaturated fatty acid from 7 days before laser photocoagulation to 7 days after laser injection. The area of lesions was quantified in flat-mounted choroids with IB4 staining 7 days after laser injection. n = 20 mice/group. *** p < 0.001.

Previously we reported that dietary intake of omega-3 long-chain polyunsaturated fatty acid (LCPUFA) reduces pathological retinal angiogenesis in oxygen-induced retinopathy [17]. Several previous studies also report protective effects of omega-3 dietary lipids and their metabolites on laser-induced CNV in rabbits and rats [18,19]. To evaluate the use of the image-guided laser-induced CNV model in the evaluation of potential treatments, we analyzed the effect of dietary LCPUFAs feed on CNV development. Mice at ~5 weeks of age were fed with either omega-6 or omega-3 LCPUFA enriched diets for 1 week before laser photocoagulation and throughout the experiment. The lesion area at 7 days after laser photocoagulation was significantly smaller in omega-3 LCPUFA-fed mice compared to omega-6 LCPUFA feed (Fig 7 and Table 4). These results confirm our earlier studies and indicate that omega-3 LCPUFA feed suppresses laser-induced CNV development and may have beneficial effects on the exudative form of AMD. In addition, optimal use of the image-guided laser system may produce consistent data that are useful in evaluation of potential pro- and anti-angiogenic treatments.

**Table 4:** Number of CNV Lesions, Mean Area CNV, SEM, SD and % Lesion Area Relative to Area of Mice on ω-6 Feed.

Feed	ω-6	ω-3
**Number of CNV lesions**	110	105
**Mean area CNV (μm^2^)**	38893.5	28960.5
**SEM**	18796.5	11896.3
**SD**	1792.17	1160.96
**% Lesion Area Relative to Area of Mice on ω-6 Feed**	100	74.5

## Discussion

The laser-induced CNV model in mice exhibits choroidal angiogenesis under conditions of burn-induced inflammation, modeling some aspects of neovascular AMD. This model produces lesions faster and more consistently than many other genetic mouse AMD *in vivo* models, such as apolipoprotein E over-expression or superoxide dismutase 1 loss in knockout mice [6,20,21], and is more easily applicable to transgenic mice to examine mechanistic pathways. Consistent laser photocoagulation can be achieved in the mouse eye with image directed laser burns, and optional OCT and FFA can also be performed using compatible components with the laser platform. With our optimized parameters for laser photocoagulation, detailed description of experimental operation and exclusion criteria, reliable and reproducible results can be generated for analysis of the labled lesion area on retinal pigment epithelium/choroid/sclera flat-mounts, as well as lesion leakage by FFA. This model is also suitable for testing and screening new anti-angiogenic drugs and other therapy for neovascular AMD.

We found that laser CNV lesions were optimum using mice of either gender weighing 15–23 g at 6–8 weeks of age. Older mice exhibit a larger and more variable CNV area, especially older female mice, which is consistent with previous reports [11,15,16]. The larger area of CNV in older female mice is suggested to be related to their high circulating levels of estrogen, which up-regulates pro-angiogenic functions of both endothelial cells and smooth muscle cells *in vivo* and promotes wound healing in both human and animal models [22–24]. Yet we observed no difference between female and male mice at 6–8 weeks of age, in contrast to a previous report showing larger CNV lesions in female mice at 5–8 weeks of age [11]. This discrepancy may be due to differences in analysis time points and fluorescent methods between the studies. In our studies, we examined the CNV lesions 7 days after laser photocoagulation with isolectin staining of dissected choroid, which differs from the previous study analyzing mice 2 weeks post laser burn with fluorescence perfusion analysis of laser-induced CNV lesions. We suggest that young adult mice of both genders are suitable for the laser-induced CNV model for testing the efficacy of new drugs, although age and gender-matched mice may be essential for specific experiments.

The laser-induced CNV model is currently the most widely used *in vivo* model for the exudative form of AMD, yet has limitations. Driven by a wound-healing reaction, the laser-induced CNV model involves high levels of acute reaction inflammation [25,26], which is not likely typical of AMD. In this model inflammatory cells initiate the angiogenic process, as depletion of either neutrophils or macrophages reduces CNV development [27–29]. In addition, major features of AMD, such as the appearance of drusen and the influence of age, are absent in the laser-induced CNV model. The model is also limited by requiring pigmented mice for photocoagulation. Nevertheless, in the absence of an aging animal model that overcomes these limitations, the laser-induced CNV model remains one of the most commonly used mouse models for AMD research. Manipulation of physiological pathways with viruses, proteins, siRNAs, shRNAs or drugs using subretinal, intravitreous, and intraperitoneal injection, as well as ingestion through feed or water is also possible in this model.

Our studies used the image guided laser system to optimize laser-induced CNV. Compared to the conventional slit lamp system, the image-guided laser system may be more convenient (Table 5). One person can induce laser burns easily as the mouse holder can be adjusted mechanically. Proficient use of the slit lamp system requires ophthalmic training and is technically challenging for a new user. In addition, no cover glass is required to convert the corneal surface to a planar surface. The laser spot with a fixed size in the image-guided laser system can be easily moved and focused mechanically instead of manually as is required using the slit lamp system. However with the image guided laser system the size of the laser spot cannot be adjusted as it can with the slit lamp system, and therefore fine adjustment to focus the laser spot for each lesion is required to properly induce the laser burn. The Micron IV image guided laser system platform has compatible OCT, FFA and electroretinography components that may be used for analysis of ocular structure and function.

**Table 5:** Comparison between Slit Lamp System and Micron IV Platform. OCT, optical coherence tomography; FFA, fundus fluorescein angiography.

Comparison Items	Slit Lamp	Micron IV
**Cover Glass**	Needed	Not Needed
**Size of Laser Spot**	Adjustable	Not Adjustable
**Movement of Laser Spot**	Manual	Mechanical
**OCT/FFA Component**	Incompatible	Compatible

Obtaining reliable and consistent results using the laser-induced CNV model requires careful experimental design and implementation, including eye integrity check, optimal laser induction, strict and consistent exclusion criteria, masking methodology, and dependable quantification techniques. Fine focusing of real-time fundus imaging with a uniform observation of nerve fibers is an essential prerequisite for consistent laser photocoagulation in the eye. Our recommendation of the laser parameters using the image-guided Micron IV laser system is a power of 240 mW and duration of 70 ms for C57BL/6J mice. Different strains require different optimizations.

These parameters for C57BL/6J mice are consistent with other researchers’ experiences that laser shots yielding the optimal CNV lesions are those with the lowest power level and shortest duration time yet still capable of rupturing Bruch’s membrane [2]. Increased power level or duration time of the laser pulse not only increases the variability of lesion area, but also damages choroidal tissue integrity, making measurements less precise. In addition, strict and consistent criteria to exclude lesions that potentially confound the experimental data are necessary for producing reliable results. Laser photocoagulation with no Bruch’s membrane rupture will yield no CNV lesion, while choroidal hemorrhage will cause lesions much larger than fellow lesions. Excluding these questionable lesions as well as outliers will improve the data reliability. Our recommended experimental parameters resulted in more than 90% of lesions that could be included and analyzed. Both laser induction and masked quantification were performed by more than one researcher to avoid subjective bias in all experiments.

Our findings provide the optimal settings and conditions to make use of the image-guided laser system for the goal of improving the consistence and reproducibility of experimental results in the laser-induced CNV model in mice for AMD research.
